# Supplementary figures and images for: Multisite imaging of neural activity using a genetically encoded calcium sensor in the honey bee
Source: PLoS Biol. 2023 Jan 31;21(1):e3001984. doi: 10.1371/journal.pbio.3001984 (PMC9917304; doi:10.1371/journal.pbio.3001984)

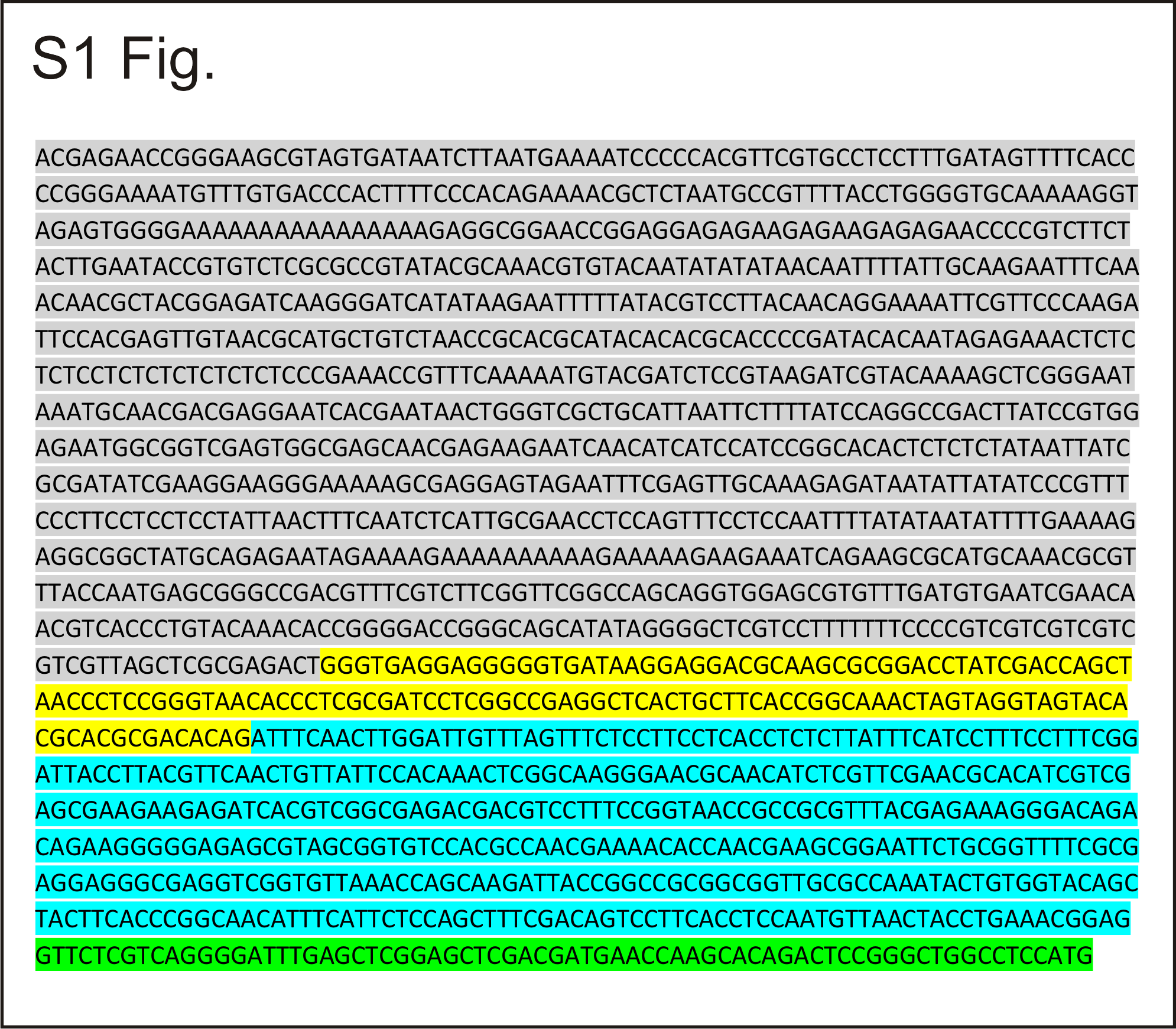

Supplement: S1 Fig — The upstream promoter region and the 5′ UTR sequence of the honey bee synapsin (syn) gene as revealed from the gene annotation release 104 (NCBI Apis mellifera Annotation Release 104; https://www.ncbi.nlm.nih.gov/genome/annotation_euk/Apis_mellifera/104/). In gray, the predicted promoter and upstream region. Different colors indicate the different annotated exons that were fused to obtain a single 5′ UTR region. (TIF) [file pbio.3001984.s001.tif]

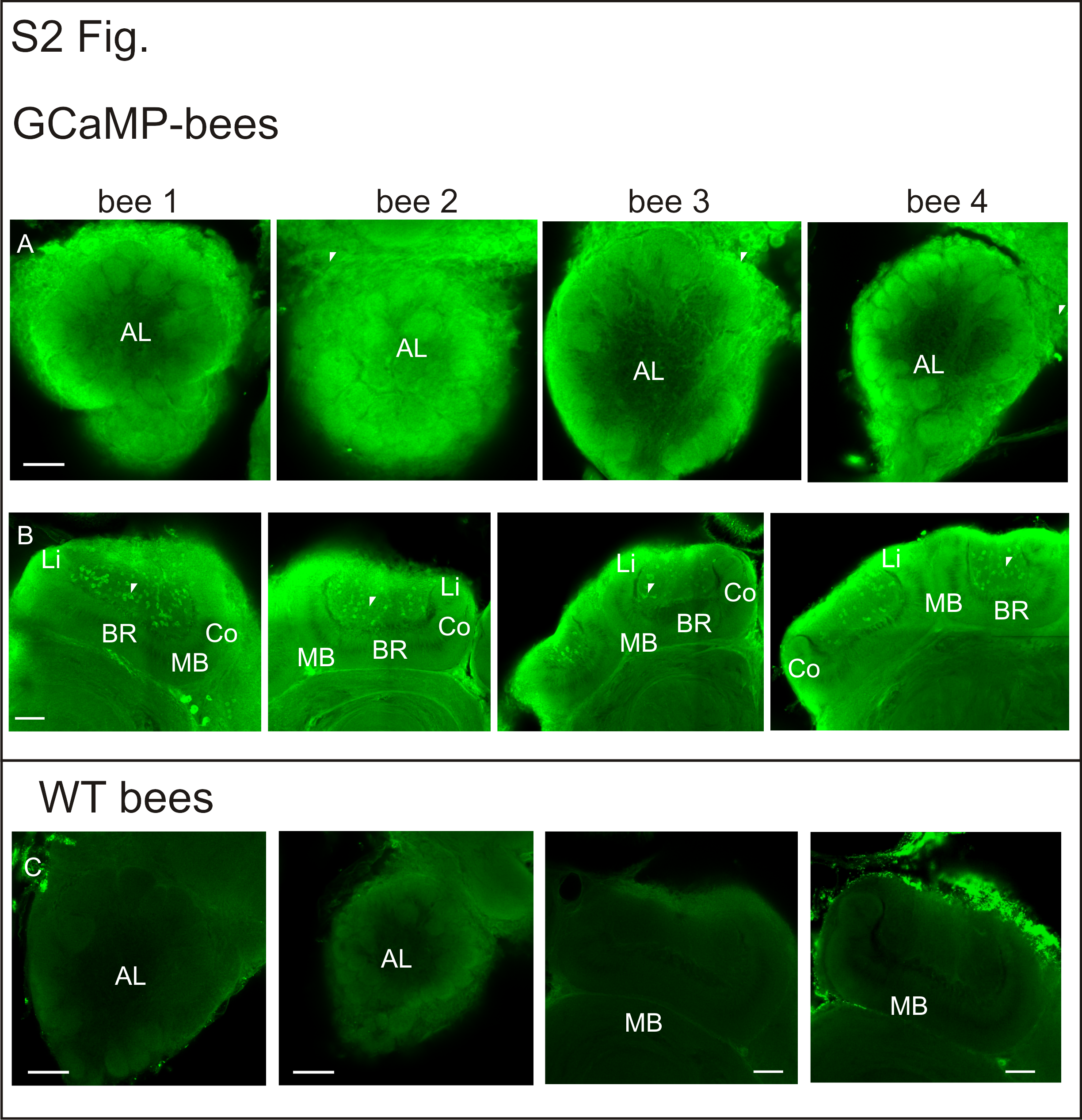

Supplement: S2 Fig — (A) GCaMP6f expression (anti-GFP in green) in the AL of different honey bees showing strong expression in the somata of projection neurons and local neurons (white arrows). (B) GCaMP6f expression (anti-GFP in green) in the mushroom bodies in different honey bees showing strong expression in somata of the Kenyon cells (in the cup of the calyces, see white arrows). Scale bar = 50 μm. AL: antennal lobe, MB: mushroom body, Li: lip, BR: basal ring, Co: collar. (C) Examples of AL and MB of WT bees showing no GCaMP6f expression. (TIF) [file pbio.3001984.s002.tif]

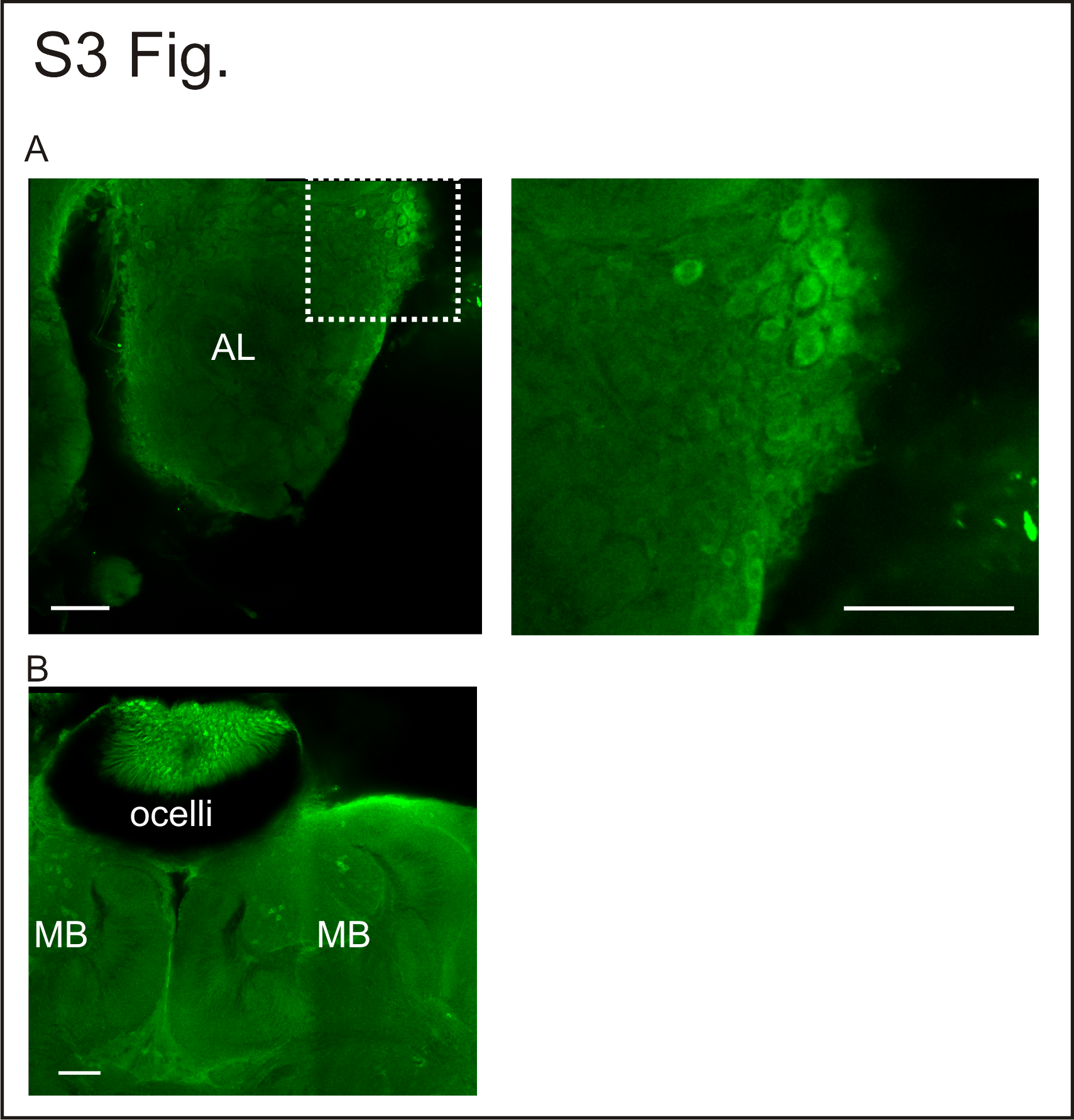

Supplement: S3 Fig — (A) GCaMP6f expression (anti-GFP in green) in the lateral cluster of local/projection neurons near the AL (left) and its zoom (right), showing very broad expression in almost all the somata. Scale bar = 50 μm. AL: antennal lobe. (B) GCaMP6f expression (anti-GFP in green) in the photoreceptors of the ocelli. MB: mushroom body. (TIF) [file pbio.3001984.s003.tif]

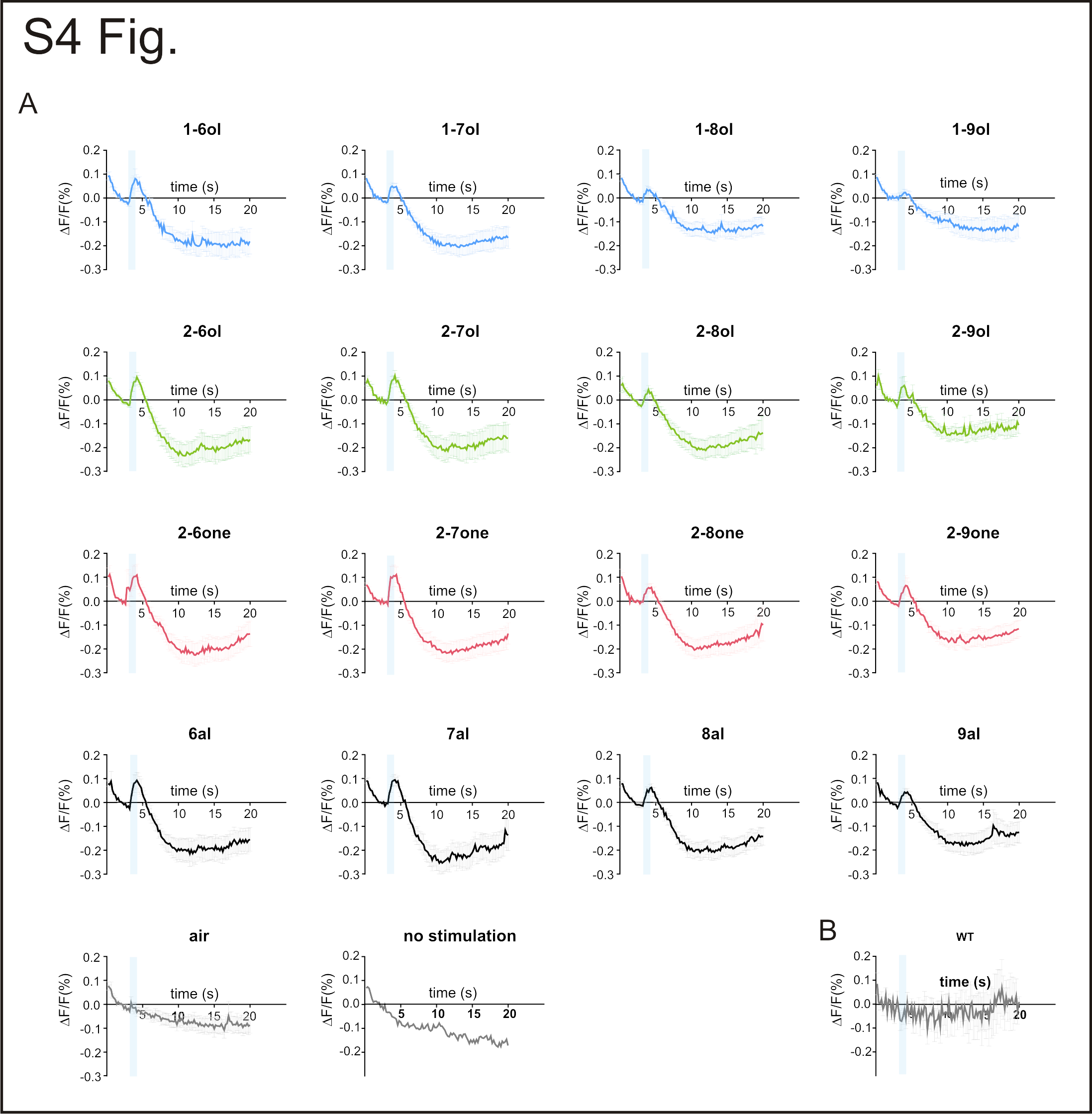

Supplement: S4 Fig — (A) Time course of odor-evoked responses (ΔF/F [%]) recorded in the AL (n = 11 honey bees) to different odorants and the air control (in gray). Time course with no olfactory stimulation (n = 2 honey bees) is also shown (in gray). (B) Time course (ΔF/F [%]) recorded in the AL of WT bees (n = 10 honey bees) following odor presentations, showing no signal in non-transgenic bees. The data underlying the graphs shown in the figure can be found in S5 Data. (TIF) [file pbio.3001984.s004.tif]

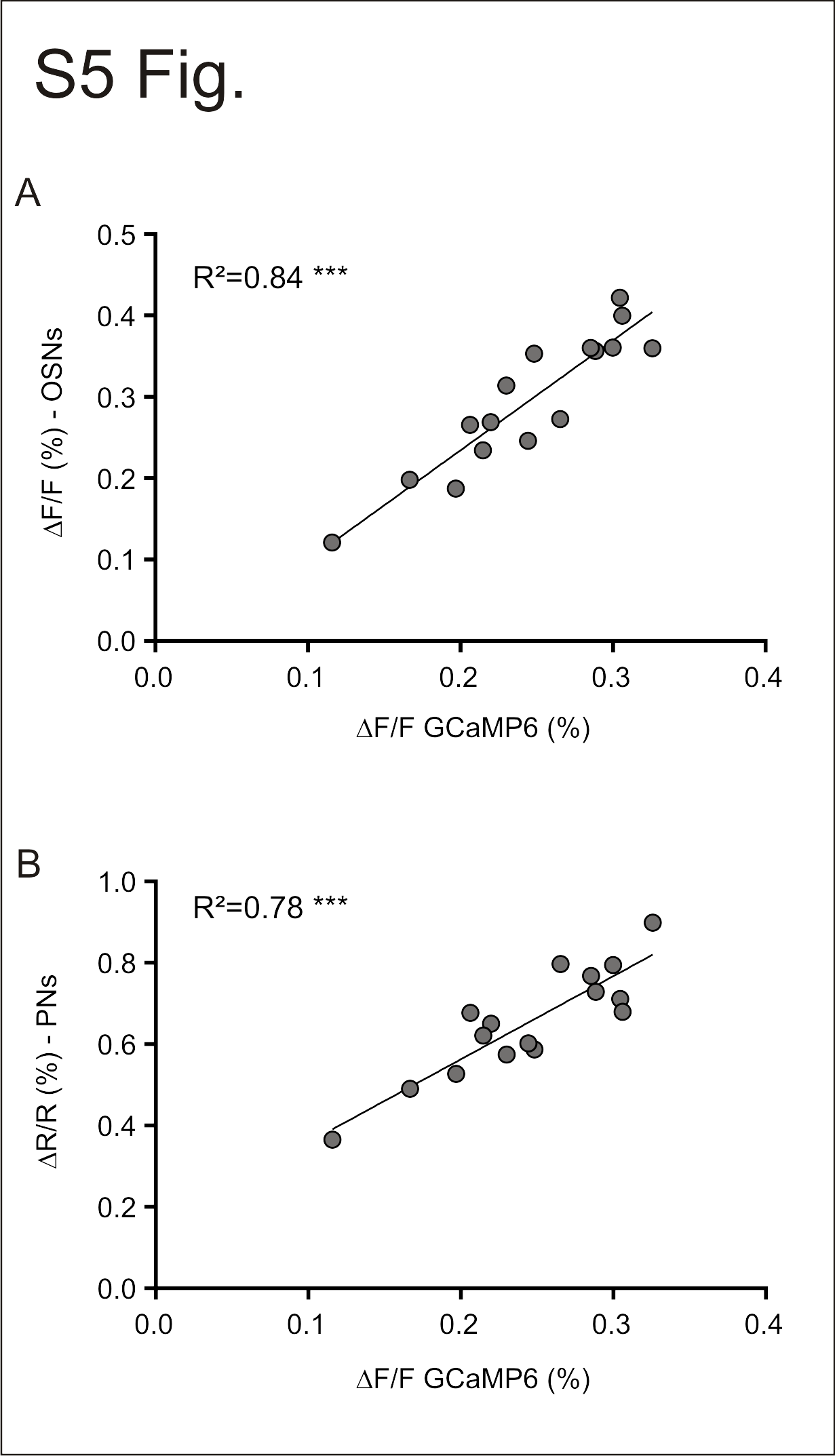

Supplement: S5 Fig — (A) Amplitude of calcium responses (ΔF/F [%]) recorded in OSNs in [46] as a function of the amplitude of calcium responses (ΔF/F [%]) recorded in the AL in this study. The linear regression shows a significant correlation (R2 = 0.84, *** p = 6 × 10^−7^). (B) Amplitude of calcium responses (ΔF/F [%]) recorded in PNs in [19] as a function of the amplitude of calcium responses (ΔF/F [%]) recorded in the AL in this study. The linear regression shows a significant correlation (R2 = 0.78, *** p = 6 × 10^−6^). (TIF) [file pbio.3001984.s005.tif]

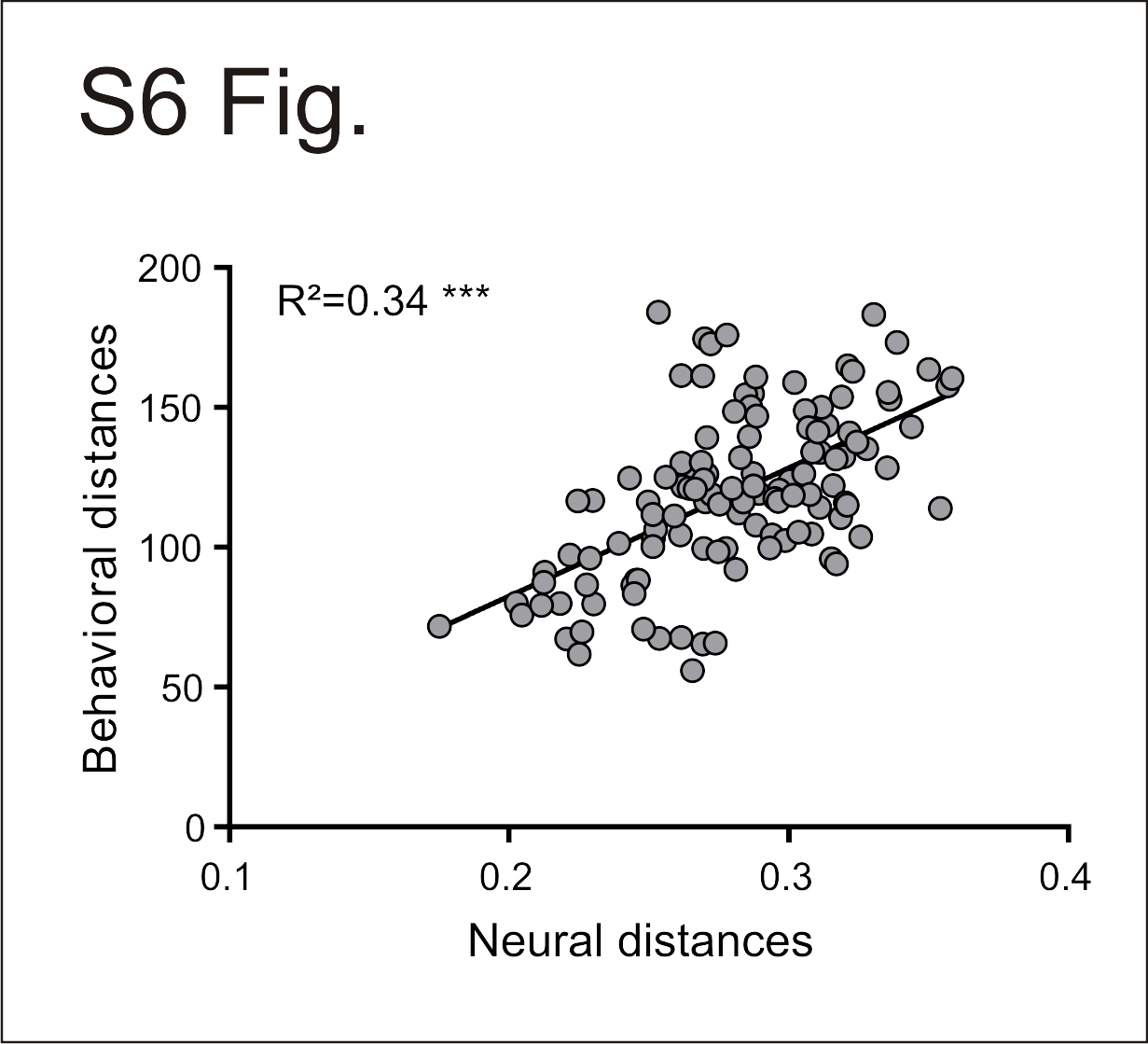

Supplement: S6 Fig — Correlation of Euclidian distances recorded in the AL in this study are highly correlated with behavioral distances recorded in [49] (R2 = 0.34, *** p < 0.001). The data underlying the graphs shown in the figure can be found in S8 Data. (TIF) [file pbio.3001984.s006.tif]

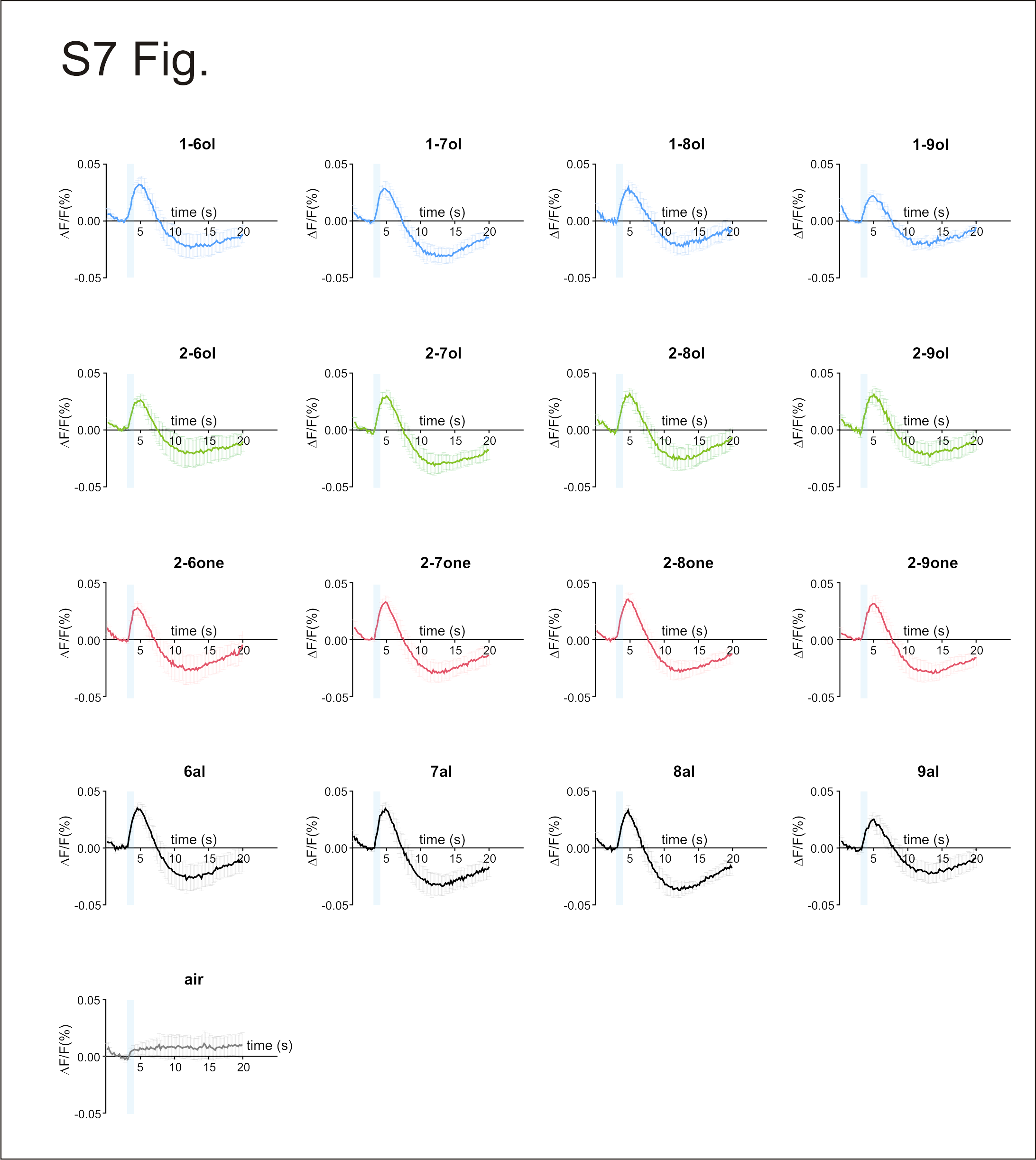

Supplement: S7 Fig — Time course of odor-evoked responses (ΔF/F [%]) recorded in the LH (n = 8 honey bees) to the different odorants and to the air control (in gray). The data underlying the graphs shown in the figure can be found in S6 Data. (TIF) [file pbio.3001984.s007.tif]

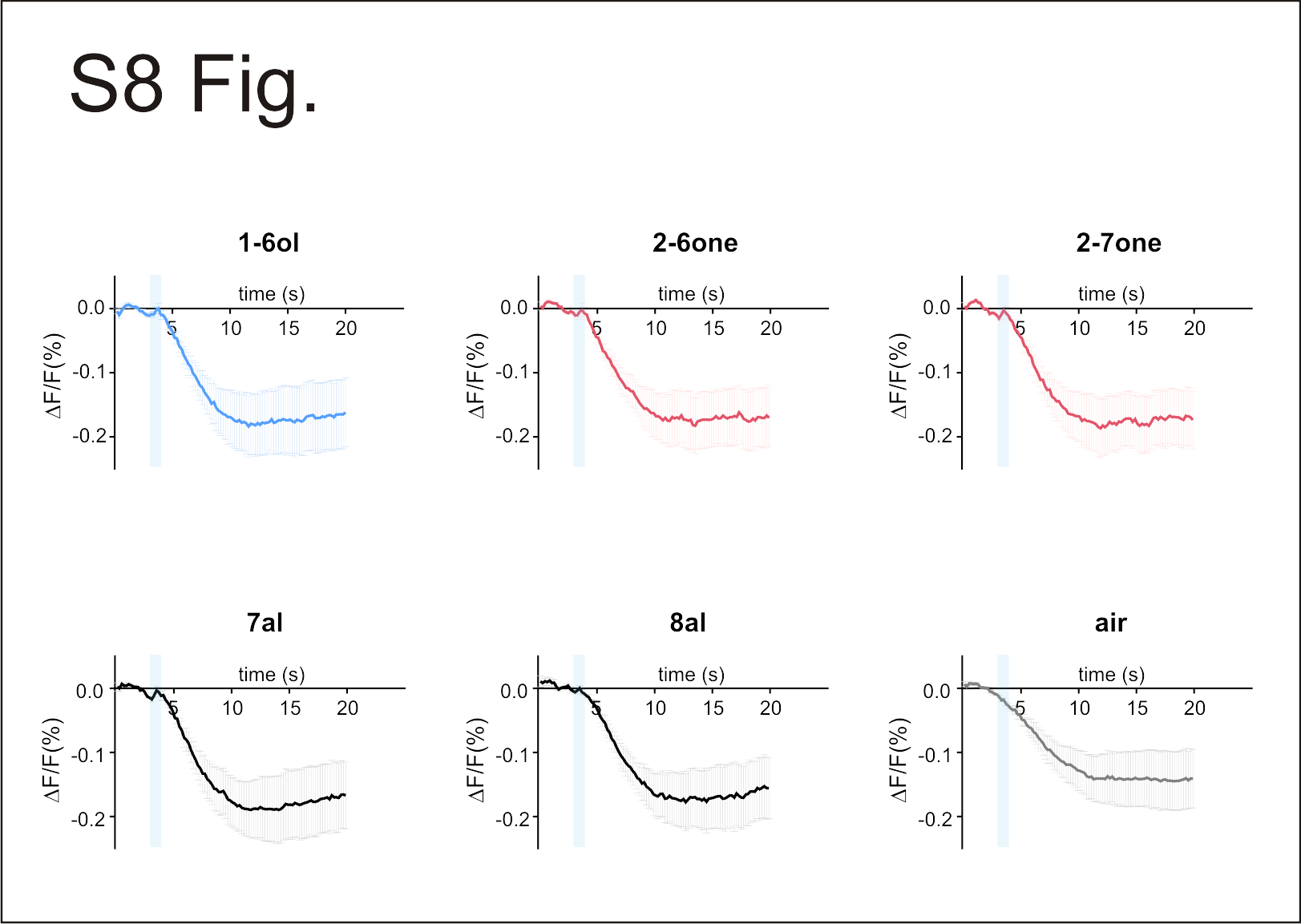

Supplement: S8 Fig — Time course of odor-evoked responses (ΔF/F [%]) recorded in the MB (n = 6 honey bees) to the different odorants and to the air control (in gray). The data underlying the graphs shown in the figure can be found in S7 Data. (TIF) [file pbio.3001984.s008.tif]
